# Decreased IL7Rα and TdT expression underlie the skewed immunoglobulin repertoire of human B-cell precursors from fetal origin

**DOI:** 10.1038/srep33924

**Published:** 2016-09-23

**Authors:** Magdalena B. Rother, Kristin Jensen, Mirjam van der Burg, Fleur S. van de Bovenkamp, Roel Kroek, Wilfred F. J. van IJcken, Vincent H. J. van der Velden, Tom Cupedo, Ole K. Olstad, Jacques J. M. van Dongen, Menno C. van Zelm

**Affiliations:** 1 Department of Immunology, Erasmus MC, University Medical Center Rotterdam, The Netherlands; 2 Department of Medical Biochemistry, Oslo University Hospital, Norway; 3 Volvat Medical Center, Oslo, Norway; 4 Center for Biomics, Erasmus MC, University Medical Center Rotterdam, The Netherlands; 5 Department of Hematology, Erasmus MC, University Medical Center Rotterdam, The Netherlands; 6 Department of Immunology and Pathology, Central Clinical School, Monash University, Melbourne, Victoria, Australia

## Abstract

Newborns are unable to mount antibody responses towards certain antigens. This has been related to the restricted repertoire of immunoglobulin (Ig) genes of their B cells. The mechanisms underlying the restricted fetal Ig gene repertoire are currently unresolved. We here addressed this with detailed molecular and cellular analysis of human precursor-B cells from fetal liver, fetal bone marrow (BM), and pediatric BM. In the absence of selection processes, fetal B-cell progenitors more frequently used proximal V, D and J genes in complete *IGH* gene rearrangements, despite normal Ig locus contraction. Fewer N-nucleotides were added in *IGH* gene rearrangements in the context of low TdT and XRCC4 expression. Moreover, fetal progenitor-B cells expressed lower levels of IL7Rα than their pediatric counterparts. Analysis of progenitor-B cells from IL7Rα-deficient patients revealed that TdT expression and N-nucleotides additions in Dh-Jh junctions were dependent on functional IL7Rα. Thus, IL7Rα affects TdT expression, and decreased expression of this receptor underlies at least in part the skewed Ig repertoire formation in fetal B-cell precursors. These new insights provide a better understanding of the formation of adaptive immunity in the developing fetus.

During the second trimester of human fetal development, B cells are generated in the liver and bone marrow (BM), providing the neonate with a diverse immunoglobulin (Ig) repertoire. Still, antibody responses towards certain antigens (e.g. tetanus or diphtheria toxoid) are impaired in neonates, and the ability to respond is only acquired with age^1^,^2^,^3^. Various processes can underlie this initial inability to mount such responses, including a “pre-mature” diversity of the Ig repertoire.

B-cell development has been extensively studied in human postnatal BM, where 5 distinct stages can be identified. In the early pro-B and pre-BI cell stages, D to J gene rearrangements and V to DJ gene rearrangements are induced by the lymphoid-specific recombination activating gene proteins 1 and 2 (Rag1 and Rag2) in the Ig heavy chain (*IGH*) locus^4^,^5^. Subsequently, the cells induce V to J gene rearrangements in their Ig light chain loci (*IGK* or *IGL*) as pre-B-II small cells and the complete Ig molecule is selected for functionality at the immature-B cell stage^6^,^7^. The Rag-induced double stranded DNA breaks activate cell cycle checkpoint protein ATM and the non-homologous end joining (NHEJ) pathway^8^,^9^,^10^, which enhances diversity in the junctional region that encodes the antigen-binding complementary determining region (CDR)3. A central player is the lymphoid-specific deoxynucleotidyl transferase protein (TdT) that randomly adds N-nucleotides to the junction^11^,^12^,^13^.

Several studies have addressed the Ig gene repertoire in human fetuses, and reported restricted repertoires due to shorter CDR3 regions in *IGH*. This restriction was the result of limited N-nucleotides in the junctions and more frequent usage of the relatively short Dh7-27, Jh2 and Jh3 genes^1^,^2^,^14^,^15^,^16^,^17^,^18^,^19^,^20^,^21^,^22^,^23^. Finally, the Dh-proximal Vh6-1 and Vh1-2 genes appear to be more frequently used in fetal B cells^1^,^24^,^25^,^26^,^27^.

Similar skewing of the *IGH* gene repertoire has been observed in mouse embryos^14^,^15^,^18^,^19^. Embryonic B-cell progenitors in the mouse liver have been shown to generate a separate B-cell lineage, and can develop independent of functional IL7R signaling^28^,^29^. Postnatal B-cell development in IL7R-deficient mice is completely blocked due to defects in proliferation and survival^29^,^30^. Furthermore, IL7R-deficient progenitor-B cells are impaired in the capacity to rearrange DJh-distal Vh genes^31^. In contrast to mouse, human B cells can develop in the absence of IL7R signaling^32^,^33^. However, the Ig gene repertoire in progenitor-B cells of IL7Rα-deficient patients has not been studied so far. Thus, it remains unclear what underlies the skewed Ig repertoire in fetal B cells and if IL-7R signaling is involved.

We here studied the nature of Ig gene repertoire formation during human fetal B cell development in B-cell progenitors, and the role of IL7Rα in this process. Through analysis of patients with genetic defects in IL7Rα, we were able to demonstrate a direct link between IL7Rα, TdT and N-nucleotide additions in *IGH* gene rearrangements.

## Results

### Skewed Ig repertoire generation in fetal B cell precursors

**Figure 1:**
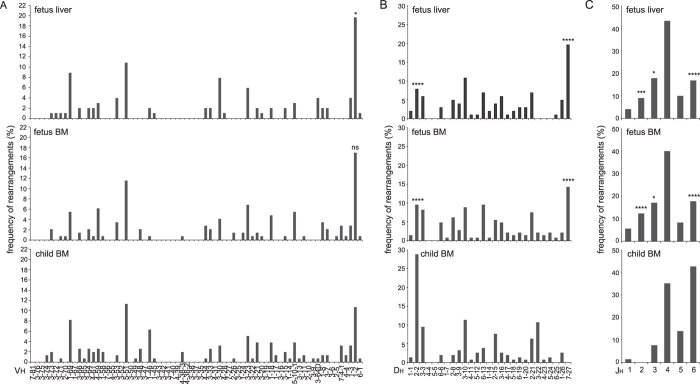
Skewed Ig repertoire generation in fetal B cell precursors. (**A**) *IGHV*, (**B**) *IGHD*, (**C**) *IGHJ* gene usage in pre-BI cells from fetal liver, fetal BM and pediatric BM. In-frame and out-of-frame rearrangements were included. 102 sequences were derived from fetal liver (3 donors), 148 from fetal BM (4 donors) and 160 from pediatric BM (5 donors). Statistics: χ2 test; *p < 0.05, ***p < 0.001, ****p < 0.0001.

To study the Ig repertoire formation in fetal development, we purified B-cell subsets from 2^nd^ trimester fetal BM and fetal liver, as well as from neonatal cord blood and pediatric BM. In line with previous observations^1^,^16^,^17^,^20^,^21^,^24^,^25^,^27^,^34^, mature B cells (CD19+CD20+CD10-IgM+IgD+) in fetal BM showed more frequent usage of Vh1-2, Dh7-27, Jh2 and Jh3 genes than pediatric B cells (Supplementary Fig. 1)^23^. This *IGH* gene usage was typical for early development, because the *IGH* repertoire in B cells from neonatal cord blood was more similar to pediatric than to fetal B cells (Supplementary Fig. 1). Moreover, CD19+CD34+CD10+ pre-BI cells derived from either fetal liver or fetal BM contained the same skewed *IGH* repertoire (Fig. 1A–C). These pre-BI cells initiate Vh to DJh gene rearrangements, but do not yet express cytoplasmic IgM and are not selected for functional *IGH* genes^35^. This was supported by analysis of only the out-of-frame and thus unproductive *IGH* gene rearrangements (Supplementary Fig. 2A–C). Approximately 1/3^rd^ of the pre-BI cells carried in-frame and thereby potentially productive *IGH* gene rearrangements (31% in fetal liver, 29% in fetal BM and 33% in pediatric BM). Thus, fetal B-cell progenitors are generated with a skewed *IGH* gene repertoire, which is similar between fetal liver and fetal BM, and does not seem to be affected by selection processes.

In contrast to *IGH*, the *IGK* gene repertoire was not different between fetal and pediatric mature B cells nor small pre-BII cells (Supplementary Figs 3 and 4). Because the vast majority of *IGK* gene rearrangements are formed in small pre-B-II cells, the formation and selection processes for the *IGK* repertoire do not seem to differ between pediatric and fetal B-cell development.

### Short Dh genes and fewer N-nucleotides in fetal B cells

**Figure 2:**
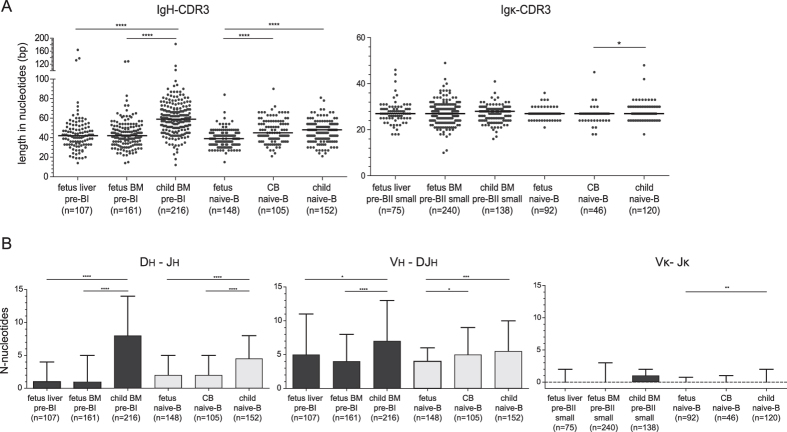
Fetal B cells have short IgH-CDR3 regions and few N-nucleotides. (**A**) CDR3 length of complete *IGH* and *IGK* gene rearrangements. Grey dots represent unique clones, black lines median values. (**B**) The median numbers of N-nucleotides in Dh-Jh, Vh-DJh and Vκ-Jκ junctions with inter-quartile range. Numbers of analyzed sequences in brackets. In-frame and out-of-frame rearrangements were included in the analysis of precursor-B cells, whereas only in-frame rearrangements for naive mature-B cells. Statistics: Mann-Whitney U test; *p < 0.05, **p < 0.01, ***p < 0.001, ****p < 0.0001.

In addition to the skewed V, D and J gene usage, *IGH* gene rearrangements in fetal naive mature B cells carried shorter IgH-CDR3 than pediatric B cells (Fig. 2A)^1^,^2^,^16^,^17^,^20^,^21^,^36^. This was mostly the result of fewer N-nucleotide additions in both the Dh-Jh and Vh-DJh junctions (Fig. 2B), as well as high usage of the short Dh7-27 gene (Supplementary Fig. 1). The IgH-CDR3 of B cells from neonatal cord blood were intermediate in size between fetal and pediatric B cells with slightly more N-nucleotides in the Vh-DJh junction than fetal B cells. Both in fetal and in pediatric tissue, the IgH-CDR3 regions in pre-BI cells were larger than in naive mature B cells. Still, fetal pre-BI carried shorter IgH-CDR3 with fewer N-nucleotides than their pediatric counterparts (Fig. 2B, Supplementary Fig. 2D,E, Supplementary Table 1).

In addition to junctional region processing, the IgH-CDR3 size is affected by the Dh gene usage as these differ greatly in size. Indeed, the Dh7-27 gene that was abundantly found in fetal B cells is the shortest of all D genes. In addition, the large Dh genes were less frequently observed in fetal than in pediatric B cells (Supplementary Fig. 5a,b). Still, as previously suggested^16^, N-nucleotide additions seem dependent on Dh gene usage. The incomplete Dh-Jh rearrangements involving the short Dh7-27 carried more N-nucleotides than rearrangements involving any other Dh gene families (Supplementary Fig. 5c). Finally, the shorter IgH-CDR3 in naive mature B cells than in pre-BI cells (Fig. 2) was associated with selection against long Dh genes. Thus, the IgH-CDR3 size seems tightly regulated through N-nucleotide additions and Dh gene usage, which differ between fetal and pediatric B-cell progenitors.

In contrast to *IGH*, the *IGK* locus does not contain D genes, and Vκ-Jκ gene rearrangements hardly contain any N-nucleotide, thus rendering small CDR3 regions. Indeed, the Igκ-CDR3 regions of the fetal, neonatal and pediatric B-cell subsets were much smaller than IgH-CDR3 and did not show overt differences between any of the subsets (Fig. 2A, Supplementary Table 2). Although the pediatric naive mature B cells tended to have more N-nucleotide additions (Fig. 2B), there was no indication of a different Igκ-CDR3 repertoire in fetus.

### B cells in BCP-ALL of presumed fetal origin show fetal Ig repertoire characteristics

Our data indicate that normal B-cell precursors from fetal tissue differ from children. To study whether these cellular and molecular differences were retained in a disease model, we analyzed the *IGH* gene repertoire in BCP-ALL samples. BCP-ALL with *MLL* rearrangements or with *TEL-AML1* fusion genes can originate *in utero*^37^,^38^,^39^,^40^. We compared the *IGH* gene repertoire of these presumed fetal BCP-ALL subgroups and with the rest, i.e. subgroups containing *E2A-PBX* translocations, *BCR-ABL* translocations, hyperdiploidy or none of these abnormalities. The average age in years at diagnosis was 0.65 ± 0.68 for *MLL* rearranged BCP-ALL and 4.85 ± 2.95 for *TEL-AML1*-positive BCP-ALL, while the non-fetal origin group aged 6.39 ± 4.18.

**Figure 3:**
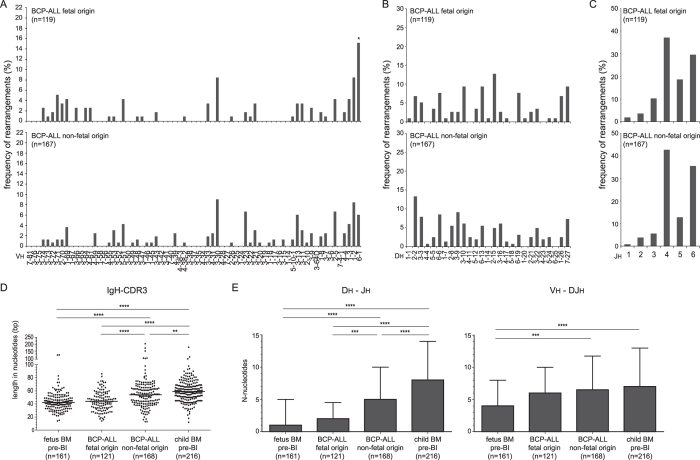
BCP-ALL of presumed fetal origin show fetal Ig repertoire characteristics. (**A**) *IGHV*, (**B**) *IGHD*, (**C**) *IGHJ* gene usage in *IGH* gene rearrangments. (**D**) IgH-CDR3 length. Grey dots represent unique clones, black lines median values. (**E**) Median numbers of N-nucleotides in Dh-Jh and Vh-DJh junctions with inter-quartile range. In-frame and out-of-frame rearrangements were included. The numbers of sequences are indicated between brackets. Statistics: χ2 (panels A/B/C), or Mann-Whitney (panels D/E); *p < 0.05, **p < 0.01, ***p < 0.001, ****p < 0.0001.

The BCP-ALL of presumed fetal origin indeed showed increased usage of the Dh-proximal Vh6-1 gene, similar to fetal B cells (Fig. 3A). In contrast, the usage of Dh and Jh genes did not differ between the BCP-ALL patients of presumed fetal and non-fetal origin (Fig. 3B,C). Still, BCP-ALL of presumed fetal origin showed a trend to less frequently use long Dh genes than the other BCP-ALL (Supplementary Figs 5a and 6a), and the IgH-CDR3 length was shorter in BCP-ALL of presumed fetal origin. The latter was due to significantly fewer N-nucleotides in Dh-Jh junctions (Fig. 3D,E, Supplementary Table 1), and equally apparent in BCP-ALL with *MLL*-rearrangements and with *TEL-AML1* translocations (Supplementary Fig. 6b,c).

Concluding, these observations in BCP-ALL confirm and extend our results that the *IGH* gene repertoire differences between fetus and child are the result of altered V(D)J recombination processes in early progenitors rather than Ig repertoire selection processes in mature B cells.

### Enrichment of early B-cell precursors in fetal liver and BM

**Figure 4:**
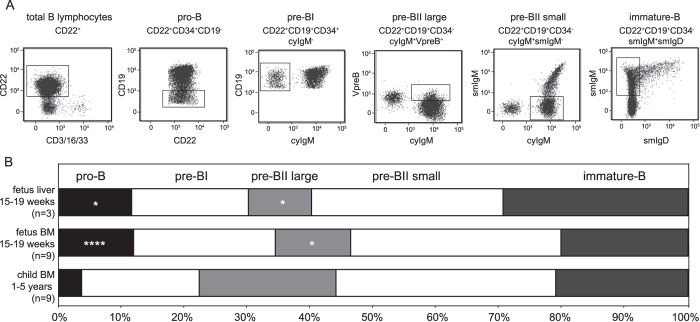
Relative increase of early precursor-B cells in fetus. (**A**) Gating strategy to define 5 precursor B-cell subsets according to previous studies^35^,^42^. (**B**) Relative distributions of the 5 precursor-B-cell subsets in fetal liver, fetal BM and pediatric BM. Statistics: Mann-Whitney U test; *p < 0.05, ****p < 0.0001.

To study the nature of the skewed *IGH* gene repertoire generation in prenatal development, we first analyzed the precursor-B cells in fetal liver and BM in more detail by flow cytometry. The relative distributions of the five major precursor-B-cell subsets in these fetal tissues were determined using a panel of membrane and intracellular markers and compared with pediatric BM (Fig. 4A)^41^,^42^. The frequencies of the pro-B, pre-BII large and pre-BII small within the total CD22+ B-cell precursors were similar between fetal liver and fetal BM. Fetal livers contained on average less pre-BI and more immature-B cells, but these differences were not significant. Both fetal tissues contained more CD34+ precursors than pediatric BM (Fig. 4B). This was mostly the result of increased pro-B cell frequencies (~12% vs 4%), and was to the expense of reduced pre-BII large frequencies (~10% vs 20%).

### *IGH* locus contraction in fetal precursor-B cells

**Figure 5:**
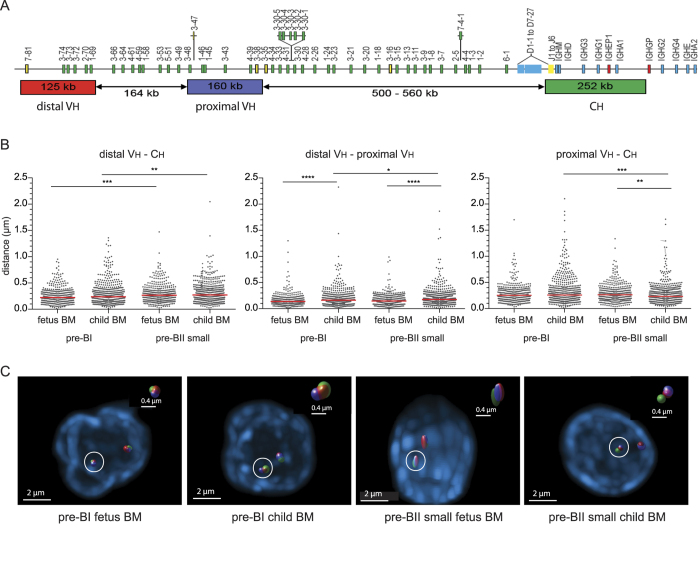
*IGH* locus contraction in fetal and pediatric B-cell precursors. (**A**) The human *IGH* locus with details of BAC clones used as 3D FISH probes^44^,^46^. (**B**) Spatial distances separating distal Vh, proximal Vh, and Ch regions in precursor-B cells from fetal and pediatric BM. Grey dots represent individual alleles, red bars median values. Per group, >200 alleles from 2 donors were analyzed. Statistics: Mann–Whitney U test; *p < 0.05, **p < 0.01, ***p < 0.001, ****p < 0.0001. (**C**) Representative images of stained *IGH* loci.

Previous studies have shown large-scale contraction of the *IGH* locus in mouse pro-B cells and the corresponding human pre-BI cells that facilitate rearrangements involving Dh-distal Vh genes^43^,^44^,^45^,^46^,^47^. Thus, the skewed Ig repertoire formation and developmental shift observed in fetus at pro-B/pre-BI stage could potentially result from inefficiently formed *IGH* gene rearrangements due to aberrant *IGH* locus topology. To study whether the *IGH* topology provided optimal conditions for V(D)J recombination, we measured spatial distances between distal Vh, proximal Vh, and Ch regions with 3D DNA FISH in precursor-B cells of fetal and pediatric BM (Fig. 5A). In line with our previous studies^44^,^46^, spatial distances between all three DNA regions were short in pediatric pre-BI cells and slightly, yet significantly, larger in pediatric pre-BII small cells (Fig. 5B,C). *IGH* locus contraction did not seem defective in fetal pre-BI cells, as all spatial distances between Ch and both Vh regions were similarly small and distances between both Vh regions were even smaller than in pediatric pre-BI cells. *IGH* locus contraction is therefore likely to facilitate rearrangement of both proximal and distal Vh gene rearrangements similarly between fetal and pediatric B-cell precursors and it does not provide further insight into the relative abundance of Vh1-2 in the fetal *IGH* gene repertoire.

### Mechanisms causing formation of skewed Ig repertoire in fetuses

To delve deeper into the mechanism responsible for generation of the different Ig repertoires between fetal and pediatric early B-cell progenitors, we performed genome-wide gene expression profiling of fetal and childhood B-cell progenitors (Supplementary Fig. 7A). The analysis was performed in fetal pro-B and pre-BI cells, which were compared to previously published childhood subsets^44^, and was focused on genes important for B-cell development and V(D)J recombination to provide insights into the molecular environment in which Ig gene rearrangements are formed.

The expression of *RAG1* and *RAG2* was similar between fetus and child for both pro-B and pre-BI cells. The genes encoding key B-cell transcription factors *E2A*, *EBF1* and *PAX5* were also highly expressed in fetal progenitors, as was *FOXO1*, which regulates *RAG* gene expression^48^,^49^. These data are in line with the diverse *IGH* gene repertoire in fetus. Furthermore, the genes encoding pre-BCR signaling components were normally expressed and did not seem rate-limiting for differentiation into pre-BII cells (Table 1).

**Figure 6:**
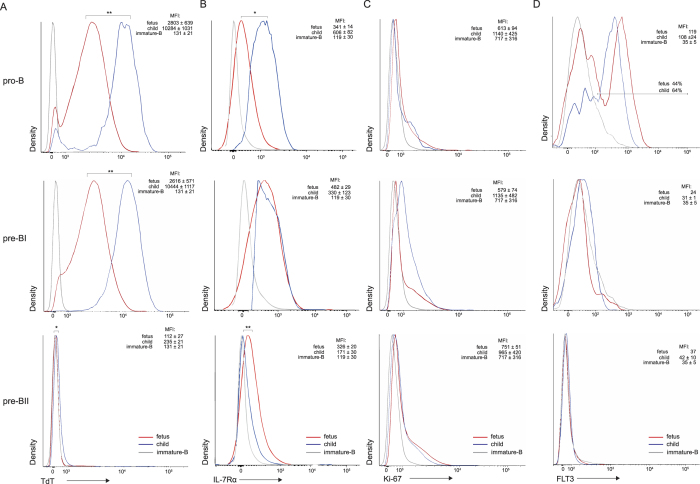
Protein expression levels in B-cell progenitors. (**A**) TdT, (**B**) IL-7Rα, (**C**) Ki-67, (**D**) FLT. Graphs represent merged data from 3–5 fetal BM (aged 15–19 weeks) and 3–6 pediatric BM samples (aged 7 years) as determined by flow cytometry analysis. Immature-B cells of pediatric BM were included as a biological negative control. In each plot, the indicated median fluorescence intensities (MFI) are shown as the averages of the individual donors with standard error of mean (SEM). Statistics were performed with an unpaired t-test on MFI values from individual donors; *p < 0.05, **p < 0.01.

However, the components of the DNA damage response pathways were downregulated in fetuses as compared to children. Among these was ATM (Table 1 and Supplementary Fig. 7b), which has a critical role in cell cycle arrest and recruitment of DNA repair factors to the broken ends^8^,^9^,^50^,^51^. Furthermore, the gene encoding XRCC4 that is part of the NHEJ pathway required for repair of Rag-induced DNA breaks^10^, was downregulated in fetal cells (Table 1 and Supplementary Fig. 7b). XRCC4 forms a protein complex with TdT, the enzyme responsible for N-nucleotide additions, and it was shown that XRCC4 promotes N-nucleotides addition by TdT^52^. Importantly, we also observed decreased expression of *DNTT*, which encodes TdT (Table 1). Additional intracellular TdT staining by flow cytometry revealed that these decreased transcripts resulted in decreased protein levels in both pro-B and pre-BI in fetuses (Fig. 6A).

In addition to DNA repair, the components of signaling pathways which increase accessibility of Ig loci for rearrangements were downregulated in fetus. IL-7R signaling affects *IGH* gene rearrangements and inhibits *IGK* gene rearrangements^46^,^53^,^54^. Fetal pro-B cells showed low expression of *IL7RA* transcripts and membrane proteins (Table 1 and Fig. 6B). Moreover, transcripts encoding the IL-7R signaling molecule JAK2 were reduced in pro-B cells, whereas transcripts of *ID2* and germline *IGK* that are normally suppressed by IL-7R signaling^55^,^56^,^57^ were upregulated (Table 1). The decreased IL-7Rα expression did not seem to affect proliferation because transcripts nor nuclear Ki-67 protein were different between fetuses and children (Table 1 and Fig. 6C). Fms-related tyrosine kinase-3 (FLT3) was also downregulated in fetal pro-B cells (Table 1 and Fig. 6D). This is the receptor for FLT3L that has synergistic effects with IL-7 on survival and proliferation of B-cell progenitors^58^.

Together, fetal B-cell progenitors differ from their pediatric counterparts in expression levels of NHEJ components and TdT, as well as surface receptors IL-7R and FLT3.

### TdT expression and N-nucleotide additions in IL7Rα-deficient patients

**Figure 7:**
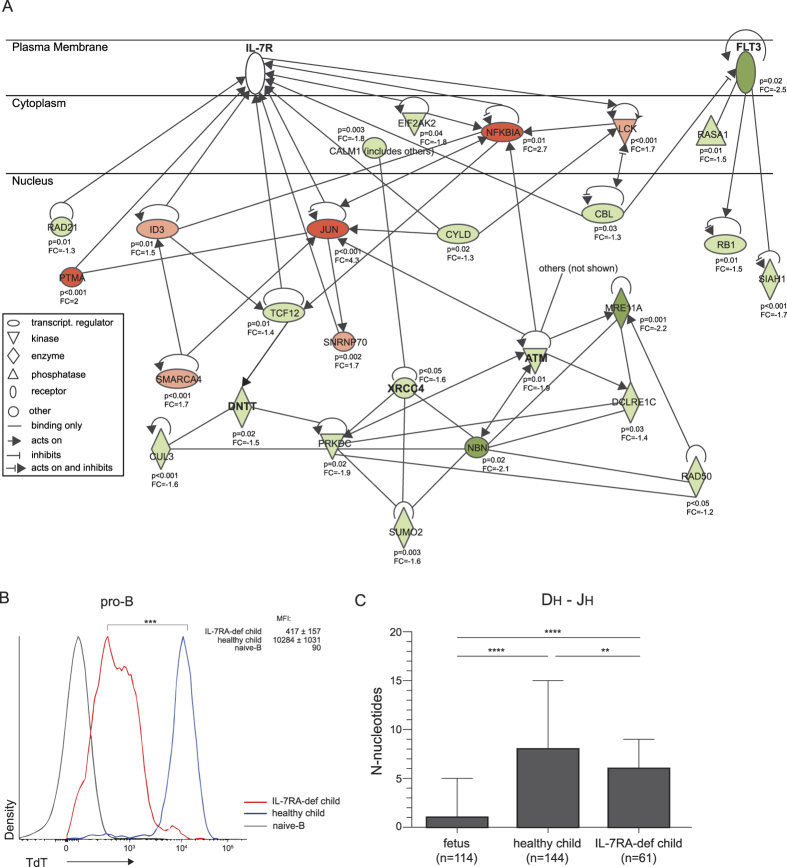
IL7Rα-regulated expression of TdT and *IGH* junctional processing. (**A**) Functional network of molecules interacting with IL-7R, FLT3, ATM, XRCC4 and TdT (*DNTT*) in pro-B cells. Upregulated (red) and downregulated (green) transcripts in fetus vs child. (**B**) TdT protein expression in cyCD79A+CD19- BM derived pro-B cells of IL7Rα-deficient children (aged 3-9 months) and pediatric controls (aged 7 years). Histograms include merged data from 3 donors for each group. In each plot, the indicated median fluorescence intensities (MFI) are shown as the averages of the individual donors with standard error of mean (SEM). Statistics were performed with an unpaired t-test on MFI values from individual donors; ***p < 0.001. (**C**) N-nucleotide additions in B cells from IL7RA-deficient and healthy children. The median numbers of N-nucleotides in Dh-Jh junctions from out-of-frame *IGH* gene rearrangements with inter-quartile range. Statistics: Mann-Whitney U test; **p < 0.01, ****p < 0.0001.

Ingenuity pathway analysis suggests that IL-7R and FLT3 signaling interact with the NHEJ pathway (Fig. 7A, Supplementary Fig. 8), and that these proteins regulate expression of TdT and N-nucleotide insertions in postnatal B-cell precursors. To confirm this relation, we tested the effects of lack of IL7R signaling: B-cell precursors from IL7Rα-deficient children. If IL7R signaling is important for TdT expression and N-nucleotide additions in postnatal B-cell precursors, IL7Rα-deficient B-cells should be defective in both. CD79A+CD19- BM-derived pro-B cells of IL7Rα-deficient children showed lower nuclear TdT protein levels than healthy children (Fig. 7B). Out-of-frame *IGH* gene rearrangements in B cells of these patients contained fewer N-nucleotides in the Dh-Jh junctions (Fig. 7C). Since these Dh-Jh junctions were generated in pro-B cells^35^, and were not subjected to selection processes, this confirms that functional IL-7R expression affects *IGH* junction processing.

## Discussion

In the present study, we showed that during fetal development a diverse Ig repertoire is formed. Still, this repertoire is skewed with regards to combinatorial diversity. IgH-CDR3 regions were shorter due to fewer N-nucleotide addition, and this was directly associated with reduced TdT and IL7Rα expression. Indeed, TdT expression and N-nucleotide additions were affected in B cells of patients with genetic defects in *IL7RA*. Thus, we here provide the first link between IL7Rα with *IGH* gene rearrangements in man, as well as a role for IL7Rα in the differences between fetal and childhood Ig gene repertoires.

The fetal Ig repertoire has been a subject of study for the past 3 decades^1^,^2^,^16^,^17^,^20^,^21^,^24^,^25^,^26^,^27^,^34^. Reported differences with postnatal B cells include V, D, and J gene usage, as well as N-nucleotide additions^1^,^16^,^17^,^20^,^34^. Still, not all findings were consistent, and several studies reported different findings^1^,^24^,^34^. We acknowledged the apparent difficulties in the assessment of the fetal Ig repertoire, and took a two-step approach to tackle these. First, we assessed the repertoire in single-sorted naive mature B cells, thereby avoiding potentially preferential PCR-based amplification of V genes. Consequently, we obtained an unbiased view of the naive B cell-repertoire^23^. Secondly, we amplified *IGH* and *IGK* gene rearrangements from early precursors that had not yet been selected for in-frame rearrangements^35^. This approach allowed us to study which of the characteristics in the mature repertoire were the result of altered V(D)J recombination processes rather than selection. Thus, we confirmed that the fetal naive mature Ig repertoire contained more Vh1-2, Dh7-27, Jh2 and Jh3 genes than pediatric B cells^1^,^16^,^17^,^20^. The high frequencies of Dh7-27 and Jh2 and Jh3 could in part be the result of absence of secondary rearrangements involving upstream Dh and downstream Jh genes. However, other than Dh7-27 usage, we did not see trends for rearrangements between upstream Dh and downstream Jh genes (not shown). Despite the high Vh1-2 usage, the Dh-proximal Vh genes were in general not more frequently used in the human fetal repertoire^24^,^25^,^26^, in contrast to the mouse^14^,^15^,^18^,^19^. Importantly, we now established that these changes in the *IGH* gene repertoire were primarily the result of altered V(D)J recombination, and not due to changes in Ig repertoire selection.

The skewed *IGH* gene repertoire in fetus did not seem to result from changes in chromatin organization as the *IGH* locus in fetal B-cell precursors was contracted to a similar extent as in children. Moreover, critical transcription factors for this process, such as E2A and Pax5 were highly expressed. The 3D spatial organization could thus support the utilization of diverse Vh genes in fetal B-cell precursors independent of genomic proximity to the Dh-Jh-Ch region^14^,^15^,^18^,^19^. It is possible that the Vh1-2, Dh7-27, Jh2 and Jh3 genes were more efficiently recombined due to their optimal RSS sequences (www.imgt.org). However, *RAG* gene expression was equally high in both fetal and pediatric B-cell progenitors, making it difficult to understand how these could be rate-limiting in fetus. Alternatively, the Vh1-2 gene promoter activity could be affected by B-cell intrinsic or extrinsic factors. A likely candidate pathway would be IL-7R signaling that is known to affect *IGH* locus activity^46^,^59^. We were unable to study the production of IL-7 in fetal or pediatric BM, but we did find evidence for reduced IL-7Rα expression and lower IL-7R signaling in fetal B-cell progenitors, indicating that specific changes in Vh gene usage could be the result of altered susceptibility to IL-7. Furthermore, we observed increased usage of the Vh1-2 gene in out-of-frame rearrangements from IL-7Rα-deficient patients (not shown). This would suggest a similar role for IL-7R signaling in human as in mouse B-cell precursors, as the latter were found to be impaired in the capacity to rearrange DJh-distal Vh genes when IL7R was defective^31^.

We found additional evidence for differences in the generation of *IGH* gene rearrangements in fetal precursors through analysis of BCP-ALL. ALL cases with *MLL* rearrangements or *TEL-AML1* translocations can arise already during fetal development. Although not all cases will actually have a prenatal origin, our comparison of these cases with other BCP-ALL subsets confirmed previously observed paucity of N and P nucleotides in *IGH* junctions in ALL cells in neonatal blood spots^60^ and low TdT transcript levels in *MLL*-rearranged BCP-ALL^61^. Furthermore, we report similar changes in the Vh, Dh and Jh gene usage as in normal fetal B-cell precursors. Considering the distinct gene expression repertoires between fetal and postnatal B-cell precursors, our observations in BCP-ALL indicate that those of *in utero* origin will be derived from distinct precursors than other ALL. Thus, it is possible that the differences between BCP-ALL subsets will not only be determined by their genetic abnormalities, but also by their origin from fetal or postnatal precursor-B cells.

Through analysis of precursor-B cells, we could defined the cause of the reported reduction in N-nucleotides in *IGH* gene rearrangements^1^,^17^,^20^,^22^,^34^. Fetal precursor B cells expressed low levels of TdT, the enzyme that inserts these during NHEJ, as well as XRCC4, the NHEJ factor that directly affects TdT function^52^. Moreover, we could link the reduced TdT expression to the low expression of IL7Rα, as our analysis of precursor B cells from IL7Rα-deficient patients revealed low TdT expression and reduced N-nucleotide additions in D-Jh junctions. This is the first evidence for a role of IL7Rα in human *IGH* gene rearrangements. In mouse models, precursor B cells in postnatal BM completely depend on IL7Rα signaling, and IL7R-deficient cells show defects in *IGH* gene rearrangements, proliferation and survival^29^,^30^,^31^. Following the identification of similar roles for human and mouse IL7Rα in preventing premature *IGK* gene rearrangements^46^,^62^, it now appears that the function of IL7Rα in human and mouse B-cell precursors is more similar than has been previously appreciated.

Despite complete absence of a functional IL-7R, IL7Rα-deficient patients showed more N-nucleotide additions in *IGH* than fetal progenitor-B cells that still expressed low levels of IL-7Rα. It is unlikely that this is due to separate effects of IL-7 and TSLP, as IL7Rα is a crucial component for the receptors of both cytokines^63^,^64^. However, FLT3L can support the development of B cells in absence of IL7Rα^28^, and acts in synergy with IL-7R to expand the hematopoietic progenitors^58^. The combined reduction in expression of both the receptor for FLT3L, FLT3, and IL7Ra in human fetal B-cell precursors might have a stronger impact on TdT and XRCC4 function than in IL7Rα-deficient patients.

A potential explanation can be derived from studies in TdT deficient mice^65^. In a competitive setting with TdT-proficient B-cell precursors, TdT-deficient B cells developed quicker and demonstrated an advantage in populating peripheral lymphoid tissues. Through low TdT expression, the developing fetus would thus be able to provide a fast wave of B cells. Decreased ATM expression likely further enhanced expansion of early progenitor-B cells in fetus with less strict processing of *IGH* junctions. Considering the low levels of TdT in fetal thymus^66^,^67^,^68^,^69^, and the low numbers of N-nucleotides in *TRB* junctions^22^, it is likely that similar mechanisms apply for T cells. In addition to relatively fast generation, the shorter CDR3 regions could equip the early waves of B cells with a relevant autoreactive repertoire to assist in clearance of apoptotic cells^36^. How the IL7Rα expression levels are regulated to mediate between a fast and skewed versus a more random repertoire remains to be determined. A likely mechanism would be the amount of FLT3L produced by stromal cells, as FLT3 signaling is shown to enhance IL7Rα expression levels^70^.

Taken together, we here showed that the skewed *IGH* gene repertoire of fetal B cells is the result of altered V(D)J recombination in progenitor B cells rather than repertoire selection. Specifically, *IGH* gene rearrangements contained fewer N-nucleotide additions due to low XRCC4 and TdT expression in fetal pro-B and pre-BI cells. Since fetal B-cell progenitors expressed low levels of IL7Rα, and TdT expression was found to be directly linked to functional IL7Rα, we here provide the first evidence for a role of IL7Rα in human *IGH* gene repertoire formation. These new insights into precursor-B cell development could contribute to a better understanding of the formation of adaptive immunity in the developing fetus.

## Methods

### Tissue sampling and flow cytometry

The use of human fetal tissue (3 fetal liver and 9 fetal BM donors) and pediatric tissue (12 pediatric BM donors), and cord blood (4 donors) was approved by the Medical Ethical Committees of the Erasmus MC and Eastern Norway, and was contingent on informed consent in accordance with the Declaration of Helsinki and the Dutch Fetal tissue act. Fetal tissues were obtained from elective abortions, and informed consent was obtained after the decision to abort was finalized. Inclusion in the study did not affect the treatment regime in any way, and did not involve any form of payment. Gestational ages of the fetuses ranged between 15 and 19 weeks, and no abnormalities were present that could potentially affect the immune-repertoire. PBMC and BM B cells from 3 IL7Rα-deficient patients aged 3-9 months were obtained from left-over material of diagnostic work-up.

All methods were carried out in accordance with guidelines and regulations of the Immunology Department, Erasmus MC.

Flow cytometric immunophenotyping and purification of B-cell subsets from pediatric BM aspirates, fetal limb BM suspensions and fetal liver homogenates were performed as described previously (see Supplementary Methods)^35^,^44^.

### Ig gene rearrangements and *IGH* locus contraction

Complete *IGH* and *IGK* gene rearrangements were amplified from DNA of bulk-sorted pediatric and fetal pre-BI and pre-BII small cells^35^,^71^, pre-BI cells of IL-7Rα-deficient patients and from umbilical cord blood mononuclear cells (Supplementary Table 3), cloned into the pGEM-T Easy vector (Promega Benelux BV, Leiden, The Netherlands) and sequenced. From RNA of single-cell sorted naive mature-B cells, cDNA was prepared and gene rearrangements were amplified in nested PCR reactions^72^. *IGH* gene rearrangements in BCP-ALL samples were determined as part of the diagnostic work-up^73^. Sequences were analyzed with ImMunoGeneTics (IMGT) information system (http://imgt.cines.fr/)^74^. All sequence data have been deposited in GenBank (accession numbers KP771015 - KP771662).

*IGH* locus contraction was studied with 3D DNA FISH as described previously (details in Supplementary Methods)^44^,^46^,^47^.

### Gene expression profiling

Microarray analysis was performed on 3 biological replicates of pro-B and pre-BI subsets that were bulk-sorted from fetal BM, and compared with previously described pro-B and pre-BI subsets from pediatric donors (details provided in Expanded View; ArrayExpress accession number E-MTAB-1422)^44^. Gene networks and canonical pathways representing key genes were identified using Ingenuity Pathways Analysis (www.ingenuity.com) following criteria of fold change >1.5 and p < 0.05 in differences in gene expression. Gene expression was confirmed with specific RQ-PCR assays (see Supplementary Methods).

### Statistics

Statistical significance was calculated by a non-parametric Mann-Whitney U test, an unpaired t-test, χ2 test or one-way ANOVA as indicated in the Figure legends. P values  < 0.05 were considered as significant. *p < 0.05; **p < 0.01; ***p < 0.001; ****p < 0.0001; ns, not significant.

## Additional Information

**How to cite this article**: Rother, M. B. *et al*. Decreased IL7Rα and TdT expression underlie the skewed immunoglobulin repertoire of human B-cell precursors from fetal origin. *Sci. Rep.* **6**, 33924; doi: 10.1038/srep33924 (2016).

## Supplementary Material

Supplementary Information

## Figures and Tables

**Table 1:** Gene expression profiling of fetal and pediatric precursor-B cells.

		Pro-B				Pre-BI			
		Mean fetus	Mean child	Fold change	p-value	Mean fetus	Mean child	Fold change	p-value
transcription factors / B-cell commitment	**E2A (TCF3)**	**226.09**	**154.98**	**1.5**	**0.02**	259.49	209.27	1.2	0.02
	ID2	121.79	43.51	2.8	0.23	**195.40**	**80.31**	**2.4**	**0.03**
	EBF1	389.42	876.11	−2.3	0.33	1182.40	1090.52	1.1	0.12
	PAX5	257.97	189.37	1.4	0.52	618.04	456.95	1.4	0.02
	HEB (TCF12)	87.07	224.12	−2.6	0.07	204.00	279.67	−1.4	0.01
	PU.1 (Spi1)	**90.22**	**57.57**	**1.6**	**<0.01**	91.75	72.83	1.3	0.06
	Foxo1	182.02	167.64	1.1	0.56	231.92	224.62	1	0.58
	IRF4	99.81	81.96	1.2	0.45	140.36	135.16	1	0.87
	IRF8	**106.55**	**48.62**	**2.2**	**0.03**	**113.48**	**74.69**	**1.5**	**0.02**
	IKZF1 (Ikaros)	86.01	151.17	−1.8	0.05	201.46	172.66	1.2	0.25
	IKZF2 (Helios)	59.77	65.55	−1.1	0.77	248.02	277.30	−1.1	0.66
	IKZF3 (Aiolos)	21.92	17.95	1.2	0.43	58.31	76.44	−1.3	0.32
V(D)J/DNA repair	RAG1	103.62	121.84	−1.2	0.47	165.91	150.29	1.1	0.39
	RAG2	113.90	209.38	−1.8	0.43	227.32	309.86	−1.4	0.05
	XRCC5 (Ku80)	186.08	554.95	−2	0.15	606.18	725.66	−1.2	0.04
	XRCC6 (Ku70)	44.06	39.20	1.1	0.50	48.62	31.89	1.5	0.09
	DNA-PKcs (PRKDC)	**60.68**	**196.41**	−**3.2**	**0.01**	**149.73**	**290.76**	−**1.9**	**0.02**
	Artemis (DCLRE1C)	**41.31**	**71.56**	−**1.7**	**0.02**	87.42	121.32	−1.4	0.03
	XRCC4	**31.30**	**80.04**	−**2.6**	**0.049**	**79.37**	**124.96**	−**1.6**	**0.049**
	XLF (NHEJ1)	51.80	49.12	−1.1	0.26	54.44	49.08	1.1	0.14
	LIG4	243.02	300.93	−1.2	0.84	746.53	472.78	1.6	0.14
	TdT (DNTT)	399.84	1693.51	−4.2	0.17	**1111.20**	**1656.59**	−**1.5**	**0.02**
	pol λ	47.36	31.85	1.5	0.15	33.68	31.84	1.1	0.52
	pol μ	**85.47**	**55.48**	**1.5**	**0.02**	74.14	58.40	1.3	0.05
	ATM	**48.57**	**166.38**	−**3.4**	**0.04**	**106.49**	**199.37**	−**1.9**	**0.01**
	MRE11A	**41.67**	**169.07**	−**4.1**	**0.02**	**96.98**	**212.86**	−**2.2**	**<0.01**
	NBS (NBN)	**43.04**	**174.16**	−**4.1**	**0.02**	**85.99**	**178.98**	−**2.1**	**0.02**
	Rad50	96.36	290.60	−3	0.15	264.57	326.31	−1.2	0.049
pre-BCR signaling	CD22	83.09	64.89	1.3	0.18	**545.90**	**295.23**	**1.9**	**<0.01**
	CD19	**117.52**	**68.81**	**1.7**	**0.01**	**138.91**	**82.83**	**1.7**	**<0.01**
	CD34	210.88	295.12	−1.4	0.42	329.68	268.36	1.2	0.48
	CD10	217.69	969.99	−4.5	0.15	**880.24**	**1275.02**	−**1.5**	**0.03**
	CD79A	150.68	139.54	1.1	0.86	**268.79**	**143.01**	**1.9**	**<0.01**
	CD79B	183.05	103.54	1.8	0.27	228.40	158.76	1.4	0.05
	VPREB1	297.75	501.40	−1.7	0.49	778.90	644.68	1.2	0.18
	λ14.1 (IGLL5)	38.86	29.63	1.3	0.10	36.88	35.34	1	0.24
	BTK	106.92	142.97	−1.3	0.50	193.35	183.15	1.1	0.64
	SYK	106.83	170.55	−1.6	0.23	208.60	219.74	−1.1	0.14
	BLNK	284.80	812.93	−2.9	0.20	696.87	672.31	1	0.68
	IGHG1	89.03	65.57	1.4	<0.01	110.02	82.20	1.3	0.01
	IGKC	137.41	54.08	2.5	0.09	313.73	192.59	1.6	0.10
	IL2RG	100.12	238.93	−2.4	0.05	174.79	155.79	1.1	0.28
proliferation/survival	Ki−67 (MKI67)	182.64	305.69	−1.7	0.39	689.30	833.39	−1.2	0.24
	IL7Rα	**44.38**	**171.98**	−**3.9**	**0.04**	169.35	150.13	1.1	0.75
	LEF1	258.38	355.16	−1.4	0.7	893.34	1002.85	−1.1	0.03
	STAT5A	80.84	67.61	1.2	0.23	89.54	77.34	1.2	0.06
	STAT5B	66.58	92.58	−1.4	0.09	85.84	90.19	−1.1	0.43
	JAK1	170.76	303.35	−1.8	0.35	419.79	366.42	1.2	0.01
	JAK2	**38.05**	**81.16**	−**2.1**	**<0.01**	39.67	76.44	−1.9	0.07
	JAK3	61.46	42.30	1.5	0.06	60.12	48.21	1.3	0.02
	TYK2	77.17	56.01	1.4	0.049	75.25	64.44	1.2	0.64
	FLT3	**34.20**	**279.61**	−**8.2**	**0.01**	64.21	157.15	−2.5	0.30
	c-KIT	**35.86**	**73.68**	−**2.1**	**0.03**	31.01	32.66	−1.1	0.02

Expression levels are shown as mean signal values. Differences in gene expression levels between fetus and child which showed p < 0.05 and fold change >1.5 are marked in bold. Statistical significance was calculated with one-way ANOVA test. Gene expression profiling was performed on pro-B and pre-BI cells purified from 3 fetal BM and 4 pediatric BM donors.
