# Supplementary material for: Decreased IL7Rα and TdT expression underlie the skewed immunoglobulin repertoire of human B-cell precursors from fetal origin
Source: Sci Rep. 2016 Sep 23;6:33924. doi: 10.1038/srep33924 (PMC5034271; doi:10.1038/srep33924)
Supplement: Supplementary Information [file srep33924-s1.pdf]

Supplementary Information

Decreased IL7Rα and TdT expression underlie the skewed immunoglobulin repertoire of human B-cell precursors from fetal origin

Magdalena B. Rother, Kristin Jensen, Mirjam van der Burg, Fleur S. van de Bovenkamp, Roel Kroek, Wilfred F.J. van IJcken, Vincent H.J. van der Velden, Tom Cupedo, Ole K. Olstad, Jacques J.M. van Dongen, and Menno C. van Zelm

Supplementary Methods

Fig. S1. Skewed *IGH* gene repertoire in fetal naive B cells.
Fig. S2. *IGHV*, *IGHD* and *IGHJ* gene usage, IgH-CDR3 length, and N-nucleotides in D_H-J_H and V_H-DJ_H junctions in out-of-frame *IGH* rearrangements in pre-BI cells from fetal liver, fetal BM and pediatric BM.
Fig. S3. *IGKV* and *IGKJ* gene usage in single-sorted naive mature-B cells from fetal and pediatric BM.
Fig. S4. *IGKV* and *IGKJ* gene usage in bulk-sorted pre-BII small cells from fetal liver, fetal BM and pediatric BM.
Fig. S5. *IGHD* gene sizes.
Fig. S6. *IGH* junction characteristic in BCP-ALL.
Fig. S7. Gene expression profiling of fetal and pediatric precursor-B-cells.
Fig. S8. Functional network of molecules interacting with IL-7R, FLT3, ATM, XRCC4 and TdT *(DNTT)* in pre-BI cells.

Table S1. *IGH* junction characteristics.
Table S2. *IGK* junction characteristics.
Table S3. Primer sequences used for analysis of complete *IGH* and *IGK* gene rearrangements.
Table S4. PCR primers and UPL FAM-labeled hydrolysis probes for expression analysis of NHEJ factor transcripts.

Supplementary References

Supplementray Methods

Fetal and postnatal tissue

Fetal liver (3 donors) and fetal BM (9 donors) were obtained from elective abortions. Gestational age was determined by ultrasonic measurement of the diameter of the skull and ranged from 15 to 19 weeks. Four blood samples were obtained from the umbilical cord vein after birth, and 12 pediatric BM samples from children aged 2-11 years[1,2]. PBMC and BM B cells from IL7Rα-deficient patients aged 3-9 months were obtained from left-over material of the diagnostic work-up. The IL-7Rα mutations all resulted in premature stop codons. Anonymized data were retrospectively analysed from routine diagnostics of children (aged 1-18 years) diagnosed with B-cell precursor acute lymphoblastic leukemia (BCP-ALL) in the Erasmus MC in the years 2004-2014.

Flow cytometric immunophenotyping and cell sorting

Flow cytometric immunophenotyping was performed as described previously[3,4]. Additional stainings were performed for expression analysis using: CD20-PB (2H7), IgM-BV510 (MHM-88), IgD-PerCP-Cy5.5 (IA6-2), CD127-APC (A019D5; all from BioLegend), CD34-FITC (8G12), CD22-PE (S-HCL-1), CD19-PE-Cy7 (SJ25C1), CD34-APC (8G12, all from BD Biosciences) and CD10-APC-C750 (HI10A, Cytognos). TdT-FITC (HT-6) and Ki-67-FITC (KI-67; both from Zebra Biosciences) were used following fixation and permeabilization (AnDerGrub). Cells were acquired on a LSRII flow cytometer (BD Biosciences) and, following exclusion of dead cells and doublets, analyzed with FACSDiva (BD Biosciences) and Infinicyt software (Cytognos). For analysis of protein expression levels, immature or mature B cells gated from the same staining that are known not to express the proteins of interest were used as negative control[5].

B cell subsets were isolated to >95% purity from pediatric BM aspirates, fetal limb BM suspensions and fetal liver homogenates as described previously[1,2]. Briefly, mononuclear cells were obtained from Ficoll density gradient centrifugation (Ficoll-Paque PLUS), and directly stained with monoclonal antibodies: CD20-PB (2H7), IgM-BV510 (MHM-88), IgD-PerCP-Cy5.5 (IA6-2, all from BioLegend), CD34-FITC (8G12), CD123-PE (9F45), CD19-PE-Cy7 (SJ25C1), CD22-APC (S-HCL-1; all from BD Biosciences), and CD10-APC-C750 (HI10A, Cytognos). Five precursor-B-cell subsets (pro-B, pre-BI, pre-BII large, pre-BII small and immature-B) were bulk-sorted[2], and processed for DNA or RNA isolation. Naive mature-B cells were detected in BM samples likely due to blood contamination, and these were single-cell sorted on a FACSAria cell sorter with FACSDiva software (BD Biosciences).

PCR-amplification and sequence analysis of complete *IGH* and *IGK* gene rearrangements

Complete *IGH* and *IGK* gene rearrangements amplified from precursor-B cells using multiplex PCR reactions[2,6], and subsequently cloned into the pGEM-T Easy vector (Promega Benelux BV, Leiden, The Netherlands). Single-cell PCR products and individual bacterial clones were sequenced on an ABI Prism 3031 XL fluorescent sequencer (Applied Biosystems, Carlsbad, CA). V, (D), J gene usage, as well as junctional region size and composition were derived from each sequence using the international ImMunoGeneTics (IMGT) information system[7].

3D immunofluorescence in situ hybridization (FISH)

3D DNA FISH of *IGH* was performed as described previously[1,8,9] with fosmid clones 761A10 and 3777B2 (BACPAC Resources, Oakland, CA), BAC clones 47P23, 101G24 (BACPAC Resources) and 3087C18 (Open Biosystems, Huntsville, AL). Probes were either directly labeled

with Chromatide Alexa Fluor 488-5 dUTP or Chromatide Alexa Fluor 568-5 dUTP (Invitrogen) using Nick Translation Mix (Roche Diagnostics GmbH); or they were indirectly labeled using DIG-Nick Translation Mix (Roche Diagnostics GmbH). Freshly sorted pre-BI and pre-BII small cells were fixed in 4% paraformaldehyde, and permeabilized in a PBS/0.1% Triton X-100/0.1% saponin solution and subjected to liquid nitrogen immersion following incubation in PBS wih 20% glycerol. The nuclear membranes were permeabilized in PBS/0.5% Triton X-100/0.5% saponin prior to hybridization with the DNA probe cocktail. Coverslips were sealed and incubated for 48hr at 37°C, washed and mounted on slides with 10 µl of Prolong gold anti-fade reagent (Invitrogen). Pictures were captured with a Leica SP5 confocal microscope (Leica Microsystems). Using a 63× lens (NA 1.4), we acquired images of ~70 serial optical sections spaced by 0.15 µm. The data sets were deconvolved and analyzed with Huygens Professional software (Scientific Volume Imaging, Hilversum, the Netherlands). The 3D coordinates of the center of mass of each probe were transferred to Microsoft Excel, and the distances separating each probe were calculated using the equation: $\sqrt{((Xa-Xb)^2+(Ya-Yb)^2+(Za-Zb)^2)}$, where X, Y, Z are the coordinates of object a or b.

Microarray analyses

The microarray analysis was performed as described previously[1]. Total RNA was used to synthetize cDNA with the Ovation Pico WTA System protocol (NuGEN). Sense strand cDNA was generated with the WT-Ovation Exon Module Version 1.0 (NuGEN), fragmented and biotinylated using the Encore Biotin Module (NuGEN). The labeled cDNA was hybridized on the GeneChip Human Exon 1.0 (Affymetrix, Santa Clara, CA, USA). The arrays were scanned using the Affymetrix Gene Chip Scanner 3000 7G and images were processed using the AGCC

(Affymetrix GeneChip Command Console Software) and the CEL files were imported into Partek Genomics Suite software (Partek, Inc. MO, USA). The normalized results were expressed as fold change. One-way ANOVA test was calculated on all arrays grouped into 4 categories, i.e. fetus pro-B, fetus pre-BI, child pro-B and child pre-BI in Partek Genomics Suite to select transcripts for clustering. Non-supervised cluster analysis was then performed on 1,257 transcripts which showed a false discovery rate (FDR) <0.05% using the Euclidean/average linkage algorithm.

Quantitative RT-PCR Analysis

A TaqMan-based RQ-PCR was used to confirm the expression levels of 5 NHEJ genes: *ATM*, *DCLRE1C* (Artemis), *NBN* (NBS), *PRKDC* (DNA-PKcs) and *XRCC4*. Total RNA was extracted from bulk sorted pre-BI cells using the miRNeasy Mini Kit (Qiagen) and reverse-transcribed with SuperScript II reverse transcriptase (Invitrogen). For cDNA amplification, 15 µl reaction mix was used with TaqMan Universal MasterMix (Applied Biosystems), 670 nM of each primer and 100 nM of FAM-TAMRA-labeled probe[10]. Primers were designed with the ProbeFinder software (Roche Applied Science) and probes were from the Universal ProbeLibrary (Roche Applied Science) (**Table S4**). Duplicate reactions were performed for each cDNA sample. Gene expression was analyzed with a StepOnePlus Real-Time PCR system and StepOne Software version 2.3 (Applied Biosystems). Cycle-threshold levels were calculated for each gene and normalized to values obtained for the endogenous reference gene *ABL*.

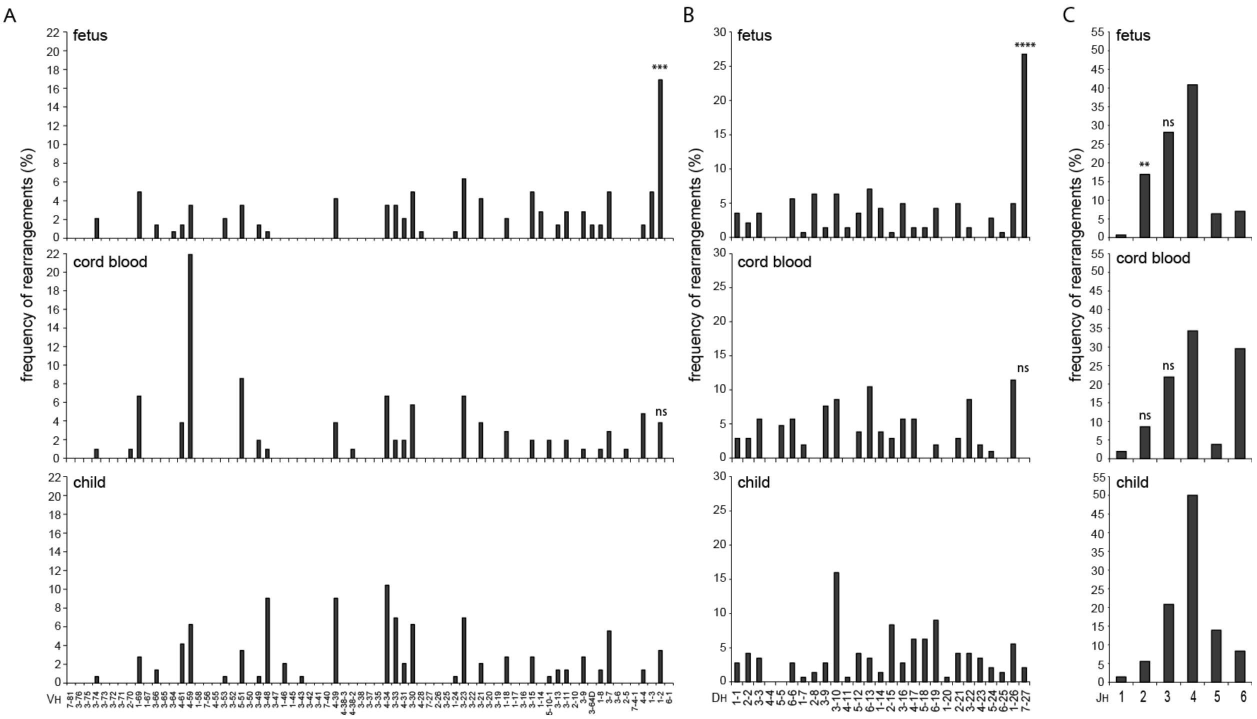

Fig. S1. Skewed *IGH* gene repertoire in fetal naive B cells. *IGHV* (**a**), *IGHD* (**b**) and *IGHJ* (**c**) gene usage in transcripts from single-cell sorted naive mature-B cells from fetal and pediatric bone marrow (BM) and in mononuclear cells from cord blood. The genes are ordered according to their genomic positions in the locus (IMGT)[7]. In total, 142 sequences were derived from 3 fetal BM samples, 105 from 4 cord blood samples and 144 from 3 pediatric BM donors. Data from individual samples showed similar patterns of gene usage. Statistical significance in gene usage between fetal and pediatric BM or cord blood and pediatric BM was determined with the $\chi 2$ test. **, p<.01; ***, p<.001; ****, p<.0001; ns, not significant.

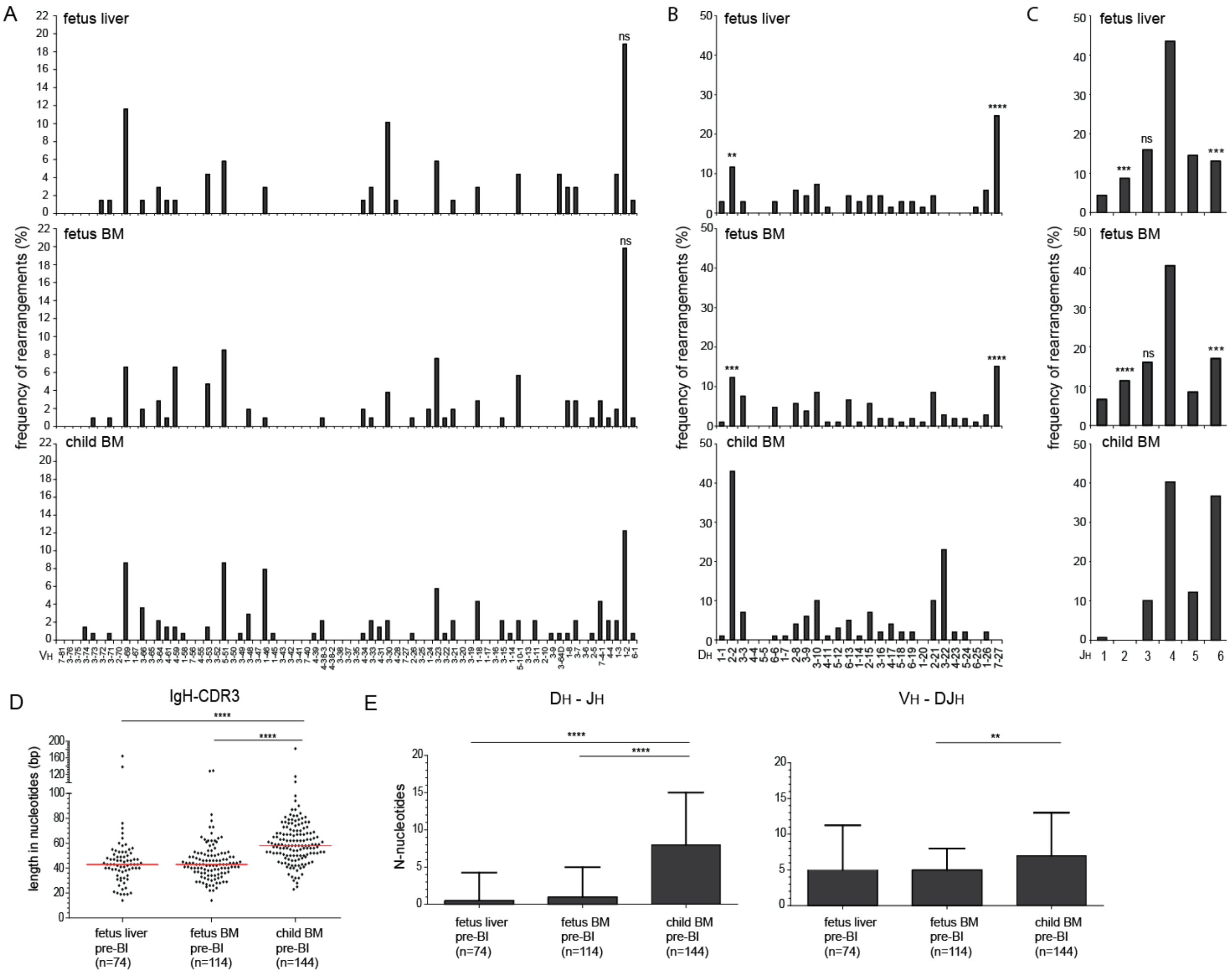

Fig. S2. *IGHV* (**a**), *IGHD* (**b**) and *IGHJ* (**c**) gene usage in out-of-frame *IGH* gene rearrangements amplified from bulk sorted pre-BI cells from fetal liver, fetal BM and pediatric BM. The genes are ordered according to their genomic positions in the locus (IMGT)[7]. Panels A-C include 69 sequences derived from 3 fetal liver samples, 106 sequences from 4 fetal BM samples and 139 sequences from 5 pediatric BM donors. (**d**) Scatter plots showing the IgH-CDR3 length in nucleotides with the red horizontal lines representing median values. (**e**) Bar graphs showing the median numbers of N-nucleotides in DH-JH and VH-DJH junctions with inter-quartile range. Numbers of sequences are depicted between brackets. Panels D-E present out-of-frame *IGH* gene rearrangements. Statistical significance was determined with the $\chi 2$ test (panels A-C) or the Mann-Whitney U test (panels D-E); **, p<.01; ***, p<.001; ****, p<.0001; ns, not significant.

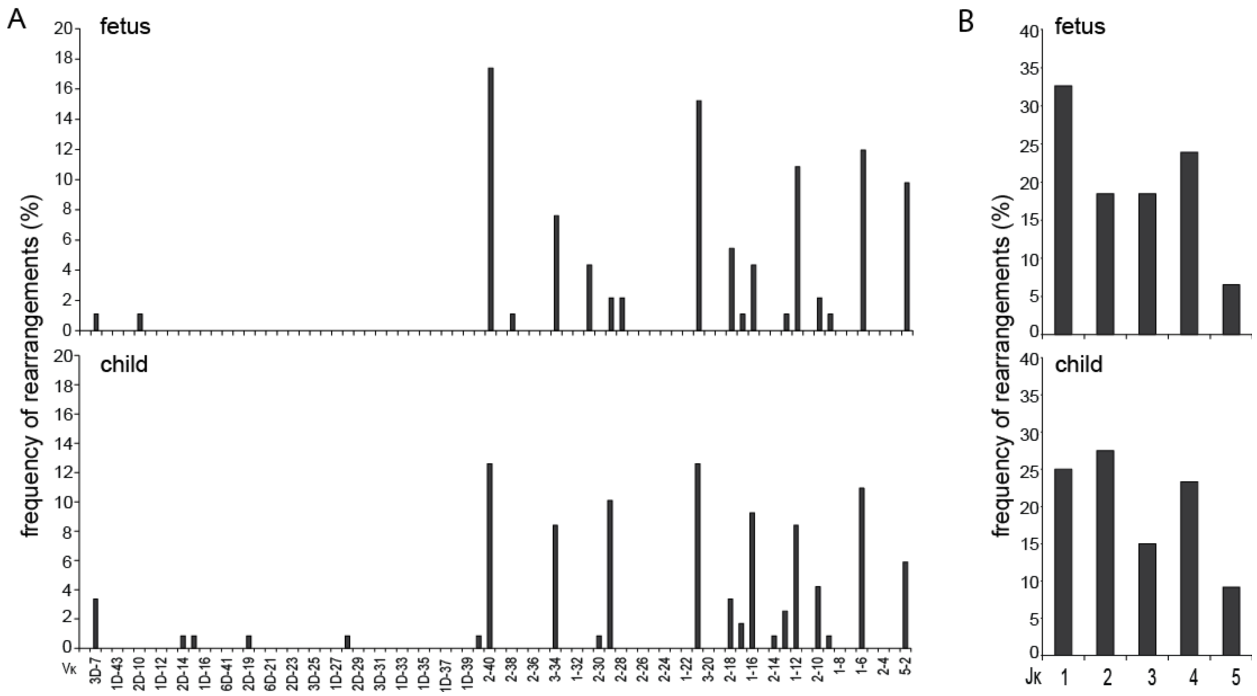

Fig. S3. *IGKV* (**a**) and *IGKJ* (**b**) gene usage in transcripts from single-cell naive mature-B cells from fetal and pediatric bone marrow (BM). The genes are ordered according to their genomic positions in the locus (IMGT)[7]. The panels include 92 sequences derived from 3 fetal BM samples and 120 sequences from 3 pediatric BM donors. Data from individual samples showed similar patterns of gene usage.

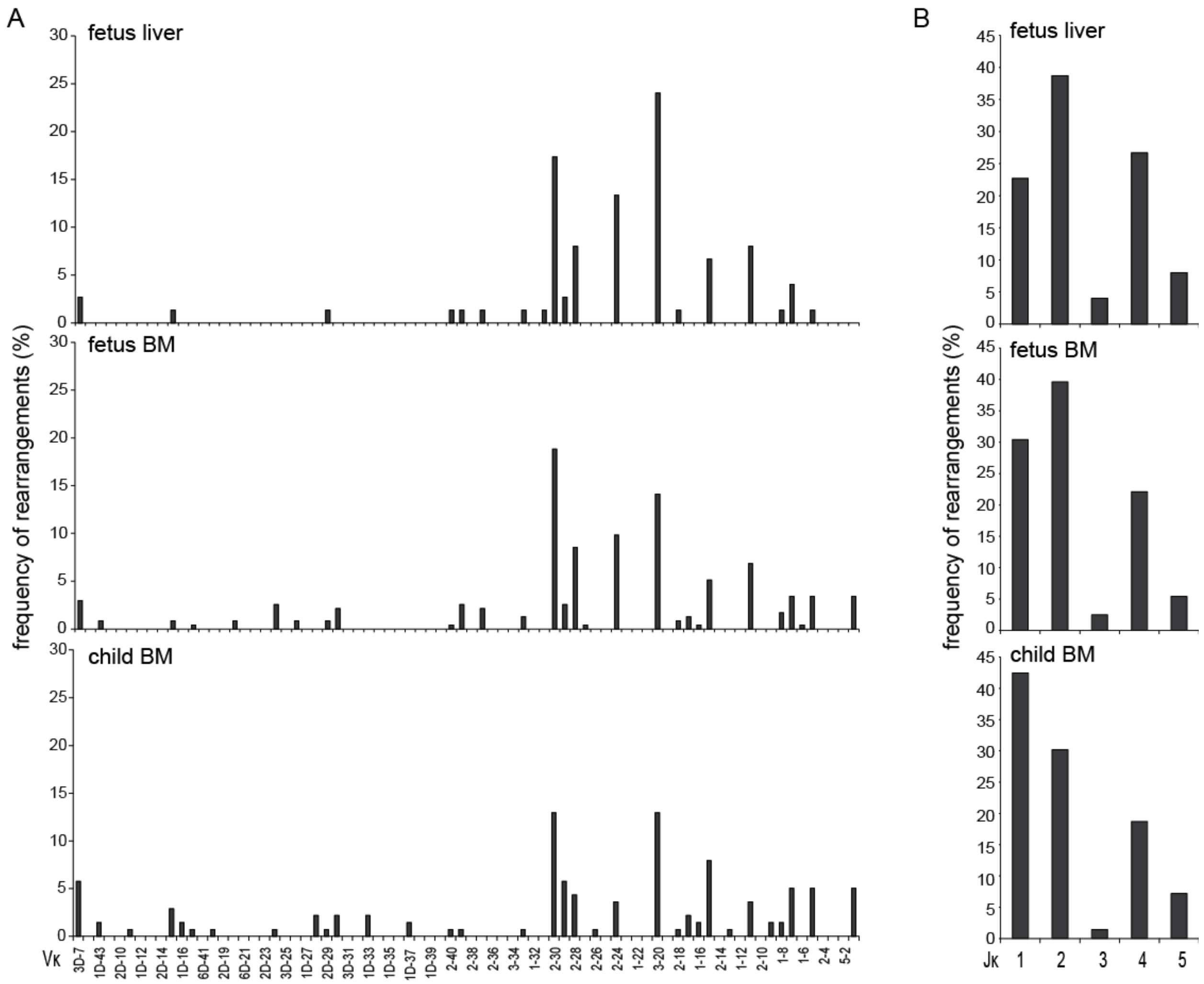

Fig. S4. *IGKV* (**a**) and *IGKJ* (**b**) gene usage in bulk sorted pre-BII small cells from fetal liver, fetal bone marrow (BM) and pediatric BM. *IGK* gene rearrangements were amplified from DNA, and both in-frame and out-of-frame rearrangements were included in the analysis. The genes are ordered according to their genomic positions in the locus (IMGT)[7]. The panels include 75 sequences derived from 3 fetal liver samples, 240 sequences from 4 fetal BM samples and 138 sequences from 5 pediatric BM donors. Data from individual samples showed similar patterns of gene usage.

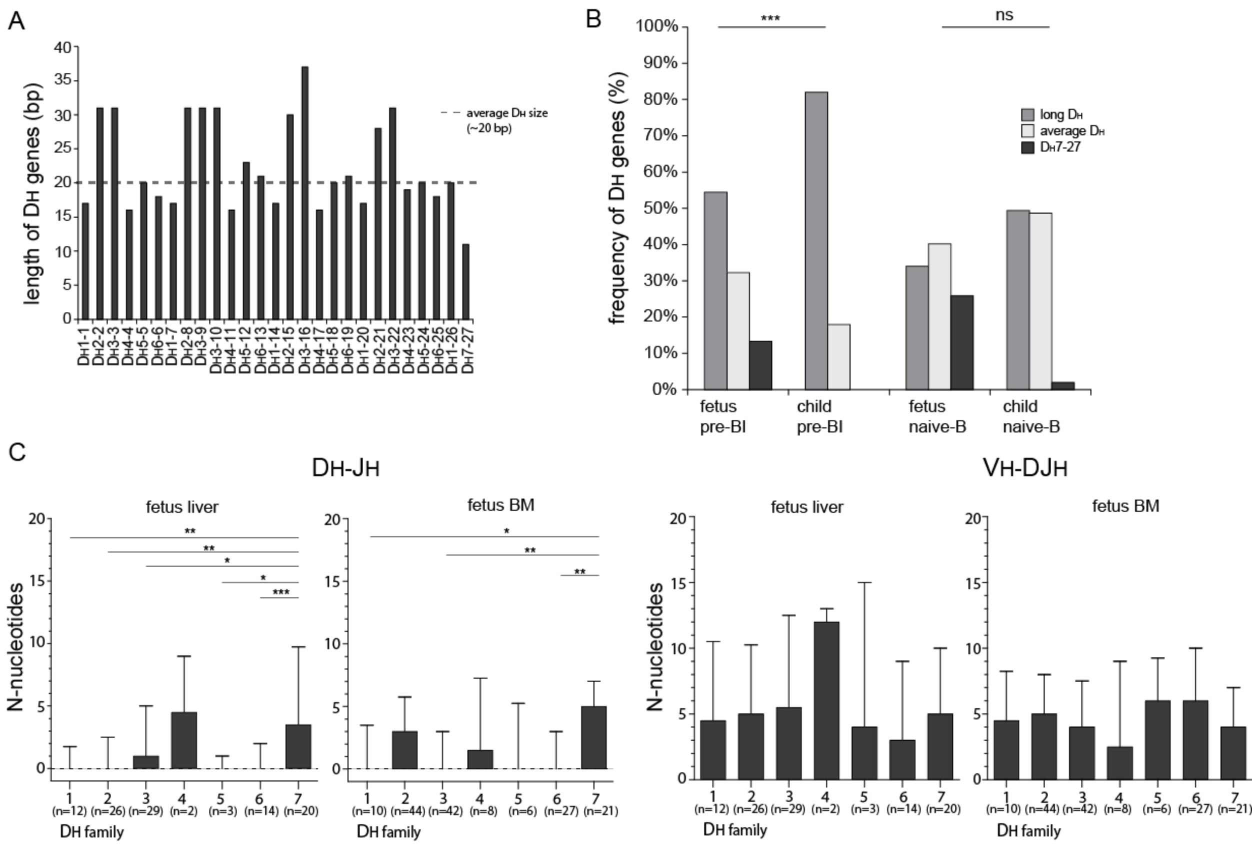

Fig. S5. *IGHD* gene sizes. (**a**) The length of germline *IGHD* genes. The majority of *IGHD* genes are ~20 nucleotides in size. DH2-2, DH3-3, DH2-8, DH3-9, DH3-10, DH2-15, DH3-16, DH2-21, DH3-22 genes are ~30 nucleotides and defined as long, whereas DH7-27 is short, being smaller than 15 nucleotides. (**b)** Relative usage of long, average and short *IGHD* genes in fetal and pediatric pre-BI and naive mature-B cells. Data include 161 sequences from 4 fetal pre-BI samples, 216 sequences from 5 pediatric pre-BI samples, 148 sequences from 3 fetal naive mature B-cell samples and 152 sequences from 3 pediatric naive mature B-cell samples. (**c**) N-nucleotides additions in DH-JH and VH-DJH junctions in pre-BI cells from fetal liver and fetal BM separated based on DH gene family usage. Bars represent medians with inter-quartile range, and include data from 3 fetal liver and 4 fetal BM donors. Numbers of analyzed sequences are indicated between brackets. Statistical significance was determined with the $\chi 2$ test (panel B) or the Mann-Whitney U test (panel C). *, p<.05; **, p<.01; ***, p<.001; ns, not significant.

Figure S6

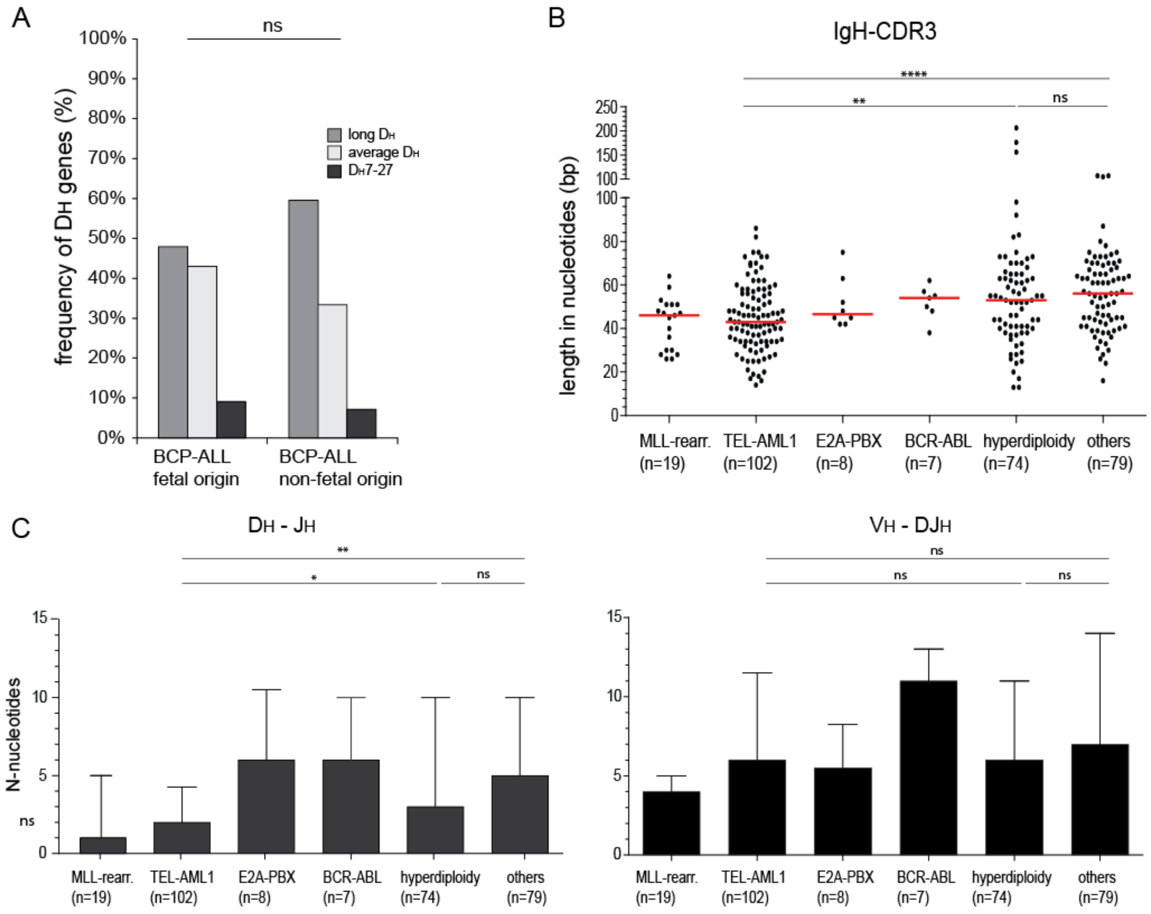

Fig. S6. *IGH* junction characteristics in BCP-ALL. (**a**) Relative usage of long, average and short *IGHD* genes in BCP-ALL subsets. BCB-ALL of fetal origin included MLL-rearranged (n=19) and TEL-AML1 positive (n=102) cases, whereas the E2A-PBX positive (n=8), BCR-ABL positive (n=7), hyperdiploid (n=74) and other cases (n=79) constituted the group of BCP-ALL from non-fetal origin. (**b**) Scatter plots showing the IgH-CDR3 length in nucleotides with red horizontal lines representing median values. (**c**) Bar graphs showing the medians number of N-nucleotides in DH-JH and VH-DJH junctions with inter-quartile ranges. The numbers of sequences are indicated between brackets. Data in all panels include in-frame and out-of-frame complete *IGH* gene rearrangements. Statistical significance was calculated with the $\chi 2$ test (panel A) or the Mann–Whitney U test; *, $p<0.05$; **, $p<0.01$; ****, $p<0.0001$; ns, not significant.

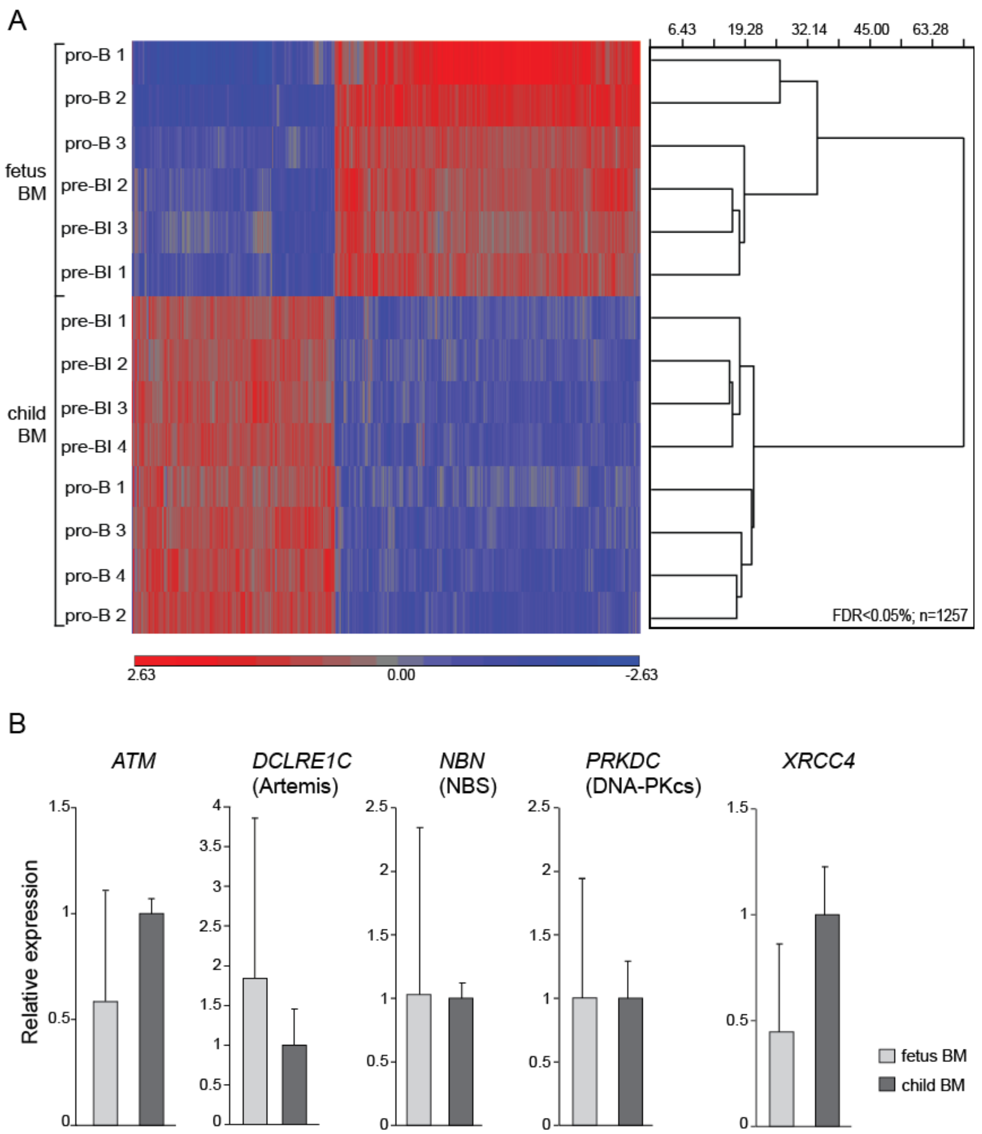

Fig. S7. Gene expression profiling of fetal and pediatric precursor-B-cells. (**a**) Non-supervised cluster analysis of the gene expression profiles from purified fetal (3 donors) and pediatric (4 donors) pro-B and pre-BI cells after one–way ANOVA test. Clustering included 1257 transcripts with a false discovery rate (FDR)<0.05% using the euclidean/average linkage algorithm. (**b**) Confirmation of gene expression levels of 5 transcripts encoding factors involved in NHEJ by real-time quantitative RT-PCR. Data was generated from bulk-sorted pre-BI cells from 2 fetal BM and 3 pediatric BM samples. Gene expression levels were normalized to the levels of *ABL*, and the values in pediatric pre-BI cells were set to 1.

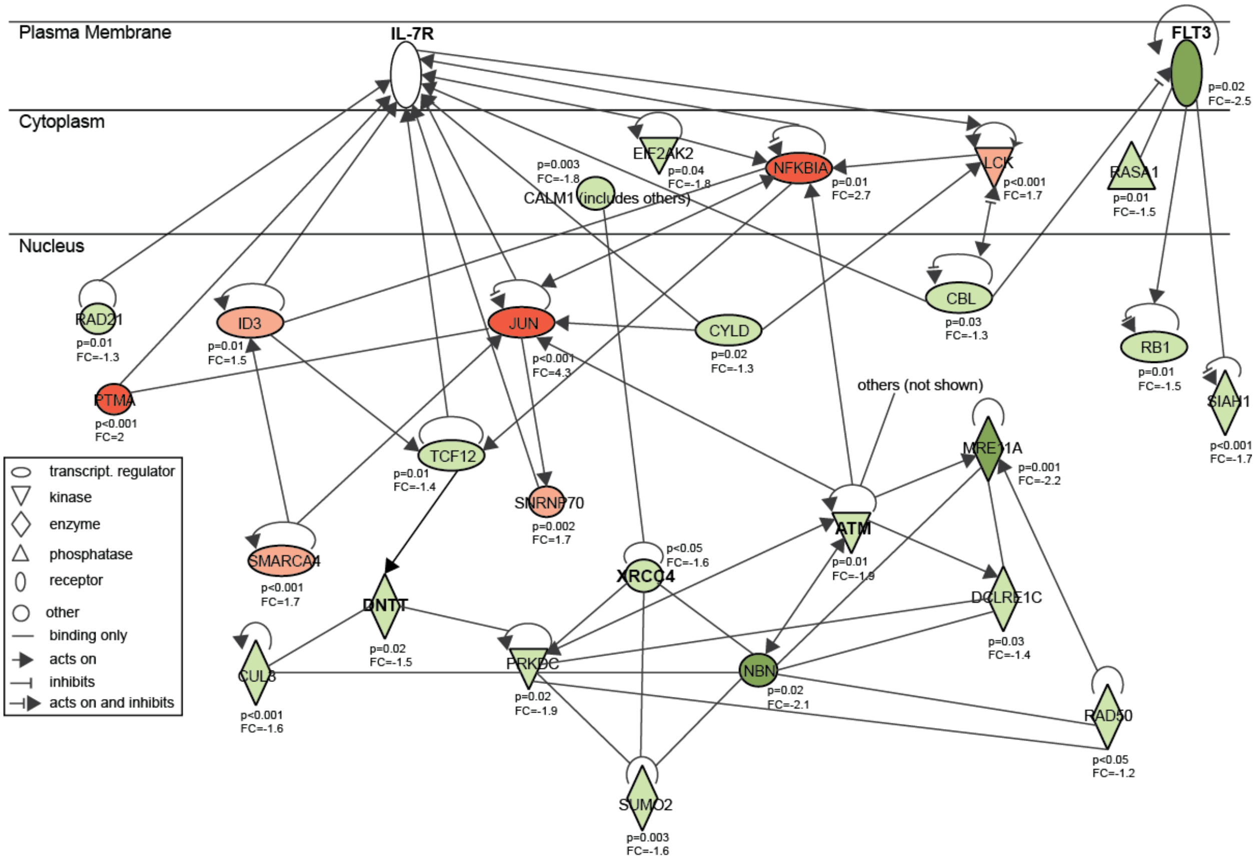

Fig. S8. Functional network of molecules interacting with IL-7R, FLT3, ATM, XRCC4 and TdT (*DNTT*) in pre-BI cells. Upregulated (red) and downregulated (green) transcripts in fetus vs child as determined by genome-wide expression profiling ($p<0.05$). Dark shades indicates fold change >2 in transcript expression, and lighter shaded fold change between <2. The interactions were identified with Ingenuity Pathway Analysis (www.ingenuity.com) starting with IL-7R, FLT3, ATM, XRCC4 and TdT (indicated in bold font).

Table S1. *IGH* junction characteristics.

	sequence no	CDR3 length (nt)	del, VH	P, VH	N, VH-DH	P, 5'DH	del, 5'DH	del, 3'DH	P, 3'DH	N, DH-JH	P, JH	del, JH
**fetus liver pre-BI out-of-frame**	74	**45.23**	1.72	0.30	8.32	0.15	4.84	4.41	0.00	**5.19**	0.08	4.15
**fetus BM pre-BI out-of-frame**	114	**45.29**	1.81	0.25	6.32	0.04	5.49	4.70	0.00	**4.26**	0.11	3.76
**child BM pre-BI out-of-frame**	144	60.56	1.84	0.32	8.98	0.22	4.99	4.29	0.03	11.19	0.15	5.49
**fetus liver pre-BI total**	107	**44.57**	1.62	0.27	7.61	0.11	5.73	5.13	0.00	**4.21**	0.07	4.23
**fetus BM pre-BI total**	161	**43.99**	1.75	0.23	5.87	0.04	5.65	4.91	0.00	**3.73**	0.13	3.60
**child BM pre-BI total**	216	59.86	1.84	0.35	8.62	0.20	4.94	4.89	0.05	10.55	0.15	5.42
**BCP-ALL fetal origin**	120	44.63	2.83	0.22	7.46	0.12	5.94	4.85	0.08	3.55	0.05	5.98
**BCP-ALL non-fetal origin**	168	55.74	2.32	0.34	9.29	0.07	5.31	4.05	0.13	7.97	0.17	4.70
**fetus BM naive-B**	148	**39.14**	1.15	0.28	4.57	0.11	5.68	4.97	0.13	**3.16**	0.13	3.23
**CB naive-B**	105	46.77	1.39	0.19	6.12	0.02	5.24	5.03	0.10	**3.25**	0.12	3.76
**child BM naive-B**	152	46.22	1.13	0.40	6.42	0.07	5.07	5.10	0.05	5.78	0.11	4.86

Significant differences in fetal *IGH* junctions as compared to childhood are indicated in bold font; Mann-Whitney U test; $p<0.05$

Table S2. *IGK* junction characteristics.

	number of sequences	CDR3 length (nt)	del, Vκ	P, Vκ	N,Vκ-Jκ	P, Jκ	del, Jκ
**Out-of-frame rearrangements pre-BII small**							
**fetal liver**	61	28.15	3.13	0.16	3.02	0.10	2.33
**fetal BM**	182	26.87	3.07	0.13	2.28	0.13	2.63
**pediatric BM**	105	27.05	3.82	0.11	2.23	0.31	2.01
**Total rearrangements pre-BII small**							
**fetal liver**	75	27.53	3.11	0.13	2.64	0.08	2.47
**fetal BM**	240	27.15	2.98	0.12	2.18	0.13	2.41
**pediatric BM**	138	27.06	3.75	0.09	2.26	0.30	2.06
**Expressed in naive B cells**							
**fetal BM**	92	27.10	2.48	0.07	**0.61**	0.09	1.37
**neonatal cord blood**	46	26.48	2.63	0.07	0.83	0.09	2.13
**pediatric BM**	120	27.63	2.88	0.08	1.44	0.08	1.31

Significant differences in fetal *IGH* junctions as compared to childhood are indicated in bold font; Mann-Whitney U test; $p<0.05$

Table S3. Primer sequences used for analysis of complete *IGH* and *IGK* gene rearrangements.

PCR type	Locus	Target	Primer	Type	Sequence (5'-3')
Ig gene rearrangements in precursor B-cells	*IGH*	VH - DJH	VH1-FR2 [a]	Forward	CTGGGTGCGACAGGCCCCTGGACAA
			VH2-FR2 [b]	Forward	GATCCGTCAGCCCCCAGGGAAGG
			VH3-FR2 [a]	Forward	GGTCCGCCAGGCTCCAGGGAA
			VH4-FR2 [b]	Forward	GATCCGCCAGCCCCCAGGGAAGG
			VH5-FR2 [b]	Forward	GGTGCGCCAGATGCCCGGGAAAGG
			VH6-FR2 [b]	Forward	GATCAGGCAGTCCCCATCGAGAG
			VH7-FR2 [b]	Forward	TTGGGTGCGACAGGCCCCTGGACAA
			JH cons [a]	Reverse	CTTACCTGAGGAGACGGTGACC
Ig gene rearrangements in naive-B cells	*IGH*	VH – DJH [c]	L-VH1	Forward	ACAGGTGCCCACTCCCAGGTGCAG
			L-VH3	Forward	AAGGTGTCCAGTGTGARGTGCAG
			L-VH4/6	Forward	CCCAGATGGGTCCTGTCCCAGGTGCAG
			L-VH5	Forward	CAAGGAGTCTGTTCCGAGGTGCAG
			Cμ CH1	Reverse	GGGAATTCTCACAGGAGACGA
Ig gene rearrangements in BCP-ALL	*IGH*	VH – DJH [d]	VH1/7-F1-CLB	Forward	TCTGGGGCTGAGGTGAAGAA
			VH2-F1-CLB	Forward	ACCTTGAAGGAGTCTGGTCCT
			VH3-F1-CLB	Forward	GGGGGTCCCTGAGACTCTC
			VH4/6-F1-CLB	Forward	GCCCAGGACTGGTGAAGC
			VH5-F1-CLB	Forward	CTGGTGCAGTCTGGAGCAG
			JH-R1-CLB	Reverse	ACCTGAGGAGACGGTGACC
Ig gene rearrangements in precursor B-cells	*IGK*	Vκ - Jκ	Vκ1 [b]	Forward	AGGAGACAGAGTCACCATCACT
			Vκ2 [b]	Forward	CTCCATCTCCTGCAGGTCTAGTC
			Vκ3 [b]	Forward	GGAAAGAGCCACCCTCTCCTG
			Vκ4 [b]	Forward	CAACTGCAAGTCCAGCCAGAGTGTTTT
			Jκ1-4 [a]	Reverse	CTTACGTTTGATCTCCACCTTGGTCCC
			Jκ5 [a]	Reverse	CTTACGTTTAATCTCCAGTCGTGTCCC
Ig gene rearrangements in naive-B cells	*IGK*	Vκ - Jκ [c]	L-Vκ1/2	Forward	ATGAGGSTCCCYGCTCAGCTCCTGG
			L-Vκ3	Forward	CTCTTCCTCCTGCTACTCTGGCTCCCAG
			L-Vκ4	Forward	ATTTCTCTGTTGCTCTGGATCTCTG
			Cκ1	Reverse	CCAGATTTCAACTGCTCATCAGA

a, [6]
b, [2]
c, [11]
d, [12]

Table S4. PCR primers and UPL FAM-labeled hydrolysis probes for expression analysis of NHEJ factor transcripts.

Target	Forward primer (5'-3')	Exon	reverse primer (5'-3')	Exon	UPL Probe #
**ABL**	GAGAAGGACTACCGCATGGA	8	GGGATTCCACTGCCAACA	9	44
**ATM**	TTTCTTACAGTAATTGGAGCATTTTG	20	GGCAATTTACTAGGGCCATTC	21	55
**DCLRE1C (Artemis)**	ACCGCAACACTCAGATCCAT	9	CAGGGTAATTTGCTCCACTGA	10	6
**NBN (NBS)**	CCTGAAAGCAGTTGAGTCCA	5	GGTTCATCAAGAGGTGGGTAAA	6	75
**PRKDC (DNA-PKcs)**	CTGGGAAGTGTCAGCTCTTTC	22	TCTTCTAAGGATATTGCTTCGTTTG	22/23	44
**XRCC4**	TGAACCCAGTATAACTCATTTTCTACA	2	TGAACCCAGTATAACTCATTTTCTACA	3	47

Supplementary References

1 Jensen, K. *et al.* Increased ID2 Levels in Adult Precursor B Cells as Compared with Children Is Associated with Impaired Ig Locus Contraction and Decreased Bone Marrow Output. *J Immunol* **191**, 1210-1219 (2013).

2 van Zelm, M. C. *et al.* Ig gene rearrangement steps are initiated in early human precursor B cell subsets and correlate with specific transcription factor expression. *J Immunol* **175**, 5912-5922 (2005).

3 Noordzij, J. G. *et al.* The immunophenotypic and immunogenotypic B-cell differentiation arrest in bone marrow of RAG-deficient SCID patients corresponds to residual recombination activities of mutated RAG proteins. *Blood* **100**, 2145-2152 (2002).

4 Noordzij, J. G. *et al.* Composition of precursor B-cell compartment in bone marrow from patients with X-linked agammaglobulinemia compared with healthy children. *Pediatr Res* **51**, 159-168 (2002).

5 Maecker, H. T. & Trotter, J. Flow cytometry controls, instrument setup, and the determination of positivity. *Cytometry. Part A : the journal of the International Society for Analytical Cytology* **69**, 1037-1042 (2006).

6 van Dongen, J. J. *et al.* Design and standardization of PCR primers and protocols for detection of clonal immunoglobulin and T-cell receptor gene recombinations in suspect lymphoproliferations: report of the BIOMED-2 Concerted Action BMH4-CT98-3936. *Leukemia* **17**, 2257-2317 (2003).

7 Alamyar, E., Duroux, P., Lefranc, M. P. & Giudicelli, V. IMGT((R)) tools for the nucleotide analysis of immunoglobulin (IG) and T cell receptor (TR) V-(D)-J repertoires,

polymorphisms, and IG mutations: IMGT/V-QUEST and IMGT/HighV-QUEST for NGS. *Methods Mol Biol* **882**, 569-604 (2012).

8 Nodland, S. E. *et al.* IL-7R expression and IL-7 signaling confer a distinct phenotype on developing human B-lineage cells. *Blood* **118**, 2116-2127 (2011).

9 Sayegh, C. E., Jhunjhunwala, S., Riblet, R. & Murre, C. Visualization of looping involving the immunoglobulin heavy-chain locus in developing B cells. *Genes Dev* **19**, 322-327 (2005).

10 van Zelm, M. C. *et al.* Human CD19 and CD40L deficiencies impair antibody selection and differentially affect somatic hypermutation. *J Allergy Clin Immunol* **134**, 135-144 (2014).

11 Tiller, T. *et al.* Efficient generation of monoclonal antibodies from single human B cells by single cell RT-PCR and expression vector cloning. *J Immunol Methods* **329**, 112-124 (2008).

12 van der Velden, V. H. & van Dongen, J. J. MRD detection in acute lymphoblastic leukemia patients using Ig/TCR gene rearrangements as targets for real-time quantitative PCR. *Methods Mol Biol* **538**, 115-150 (2009).
